# Characteristics of menstrual cycle disorder and saliva metabolomics of young women in a high-temperature environment

**DOI:** 10.3389/fphys.2022.994990

**Published:** 2023-01-13

**Authors:** MengFan Wei, GaiHong An, LiJun Fan, XueWei Chen, Chao Li, JiaJun Chen, Qiang Ma, DanFeng Yang, Jing Wang

**Affiliations:** ^1^ Department of Operational Medicine, Tianjin Institute of Environmental and Operational Medicine, Tianjin, China; ^2^ Zhongguancun Hospital, Chinese Academy of Sciences, Beijing, China

**Keywords:** high-temperature environment, women, menstrual disorders, saliva metabolomics, neurotransmitters

## Abstract

**Objective:** Menstrual disorders induced by high-temperature environments, can seriously damage women’s reproductive health and workability. The regulation mechanism underlying it is not yet to be elucidated. Saliva is an information-rich biological fluid that can reflect systemic diseases. Here, we investigated the characteristics of menstrual cycle disorders and saliva metabolomics to provide a deeper insight of the regulation mechanism of young women in high-temperature environments.

**Methods:** Women from high and normal temperature areas of China were selected and divided into two groups—high-temperature (H group) and control (C group). A questionnaire survey was conducted in summer (July) to investigate the incidence rate of menstrual disorders, characteristics of the disorders, and factors influencing the risk of these disorders in different regions. Metabolomics was applied to analyze the characteristics of the salivary metabolites and neurotransmitters in the two groups of women with menstrual disorders.

**Results:** The incidence rate of menstrual disorders was significantly higher in the H group than that in the C group (*p* < 0.05). High-temperature environment, stress, and sleep quality were identified as critical factors associated with menstrual disorders. Non-targeted saliva metabolomics identified 64 significantly different metabolites between two groups, which mainly enriched in metabolic pathways such as carbohydrate metabolism, membrane transport, digestive system, and nucleotide metabolism (*p* < 0.05). N-acetylneuraminic acid, MYO, and tyramine may be candidate markers for early diagnosis of menstrual disorders in high temperature environments. Metabolites may be involving in the acute-phase response during an inflammatory process, to affecting the reproductive system by influencing the HPA axis loop. Regulations about oocyte membrane production and the luteal functions would be exerted in menstrual disorders. Targeted metabolomics of neurotransmitters revealed increased expression of histamine (HA) and glutamine and decreased expression of 5-hydroxyindole acetic acid (5-HIAA) (*p* < 0.05).

**Conclusion:** Menstrual disorder characteristics induced by high temperature environments were specific. Anxiety, sleep quality and temperature feeling were the key factors to the menstrual disorder. endocrine regulation mechanism and inflammatory reactions might contribute to the development of menstrual disorders through influencing the formation of the follicular cell membrane.

## 1 Introduction

High temperature, dust, and noise have been identified as significant risks in certain occupational environments (Matsuda et al., 1997). Particularly, high-temperature environments are inevitable in specific industries, which are manifested by high temperature, high humidity, and strong radiation. Women have been reported to exhibit lower tolerance to high-temperature environments than men (Marsh and Jenkins, 2002; Yu et al., 2010). Because of their unique physiological structure, women are troubled by different reproductive health concerns after exposure to these risk factors. When women are exposed to such an environment, menstrual abnormalities often appear first, after which their physiological functions change, which are mainly manifested as short, prolonged, or irregular menstrual cycles and abnormal bleeding during the menstruation period (Harlow and Campbell, 2000; Rowland et al., 2002). The duration and patterns of the menstrual cycle are essential indices of reproductive health in women (Lisabeth et al., 2004). There are several factors besides high-temperature environments that may cause menstrual disorders. In fact, menstrual disorders are related to the neuroendocrine and organic pathologies of the body, such as neuroendocrine disorders, cysts, and tumors of the reproductive organs (Adams Hillard and Deitch, 2005). These are also related to psychological factors such as long-term stress, depression, and anxiety (Rafique and Al-Sheikh, 2018). Moreover, long-term poor lifestyle habits such as smoking and drinking, following an irregular diet, and poor sleep pattern can also lead to the development of menstrual disorders (Vyver et al., 2008; Bae et al., 2018). In addition, environmental changes such as long-term exposure to high concentrations of nanoparticles (Wang et al., 2018; Peng et al., 2020; Yuan Pan et al., 2020) and the long-term use of antiepileptic drugs such as valproic acid (Lakhanpal and Kaur, 2007; Verrotti et al., 2016) or antibiotic drugs such as sirolimus (Triana and López-Gutierrez, 2021) can also cause neuroendocrine disorders, which can lead to menstrual disorders. Past studies have demonstrated that, when women are exposed to high temperatures, heat exposure (HE) can result in menstrual and endocrine disorders and ovarian dysfunction in women (Dickson et al., 2018; Zheng et al., 2019), which in turn impacts women’s quality of life, increases industrial costs and reduces the operational capacity (Labyak et al., 2002; Axmon et al., 2006; Small et al., 2006). A previous study reported that approximately 13% of female soldiers had menstrual abnormalities, which affected their daily military tasks. Among the factors that cause menstrual irregularities, the outdoor thermal environment is a critical one (Powell-Dunford et al., 2003). The incidence rate of menstrual disorders in women working in a hot and humid environment, for example, in textile factories, reached nearly 40% as per a study (Ya et al., 2000). Animal experiments showed that long-term HE significantly increased the rates of estrous cycle disorders in female rats, wherein the organ coefficient of the uterus increased, local cell proliferation occurred, and reproductive function was damaged (An et al., 2020). Roth reported that heat stress in summer reduced the pregnancy rate of cows and affected their reproductive ability (Roth, 2017). These results together indicated an increased risk of menstrual disorders in women in a high-temperature environment, which affects their reproductive health.

The prediction results of the United Nations Intergovernmental Panel on Climate Change indicated that the frequency and intensity of heat stress in summer will continue to increase with time (Izrael et al., 2007). Therefore, issues related to female reproductive health among outdoor workers and women working in special industries (such as the female military or steel mills) should be addressed. However, the mechanism through which exposure to high temperatures causes menstrual disorders has rarely been reported. Saliva is an information-rich biological fluid that can reflect systemic diseases, screen various diseases, and be collected non-invasively, conveniently, safely, and economically (Zhang et al., 2012; Kaczor-Urbanowicz et al., 2017; Ueda et al., 2020). Specific changes in the saliva metabolism may lead to the development of periodontal diseases and oral cancer. Importantly, metabolites in the blood can enter the saliva through extracellular, intracellular, or paracellular pathways, including through passive diffusion or active transport in the salivary glands or gingival sulcus (Spielmann and Wong, 2011). Therefore, saliva metabolites may provide a window for the use of other parts of the body in the early detection of human diseases.

For this purpose, this study aimed to conduct a cross-sectional survey on the menstrual conditions of women working in the same occupational category, which can be classified as the high-temperature (H group) and control (C group) groups in order to clarify the degree of influence that high-temperature environments have on women’s menstruation cycle, the characteristics of menstrual disorders, and factors affecting the disorders. Metabolomic studies were conducted to analyze the effects and changes of a high-temperature environment on the salivary metabolites and serum neurotransmitters in women to identify the key metabolites that possibly contribute to the development of menstrual disorders as well as to explore the regulatory pathways involved in the possible involvement of these key metabolites in menstrual disorders. This study is aim to provide a theoretical basis for a non-invasive and convenient menstrual disorder monitoring system and an early warning technology as well as to obtain a deeper understanding of the mechanism of women’s menstrual disorders caused by high-temperature exposure.

## 2 Materials and methods

### 2.1 Study subjects

In this study, 125 women (aged 22 ± 3 years) with a body mass index of 20.83 ± 2.6 kg/m^2^ who were working in the same occupation and had lived in the local area for more than half a year were recruited from two different regions of China. The recruited women had no smoking or drinking habits and all had the same daily working hours (7 h), exercise intensity (exercise HR:100–140 beats/min, more than 30 min/day), and diet structure (protein: 11%–13%, fat: 20%–30%, carbohydrate: 55%–65%). The northern region of China is located at 43.88°N latitude (Jilin province), which is in the northern temperate zone, and the southern region is located at 23.05°N latitude (Guangdong province), which belongs to the tropical zone. Women from the northern region (average annual temperature 0°C–10°C, summer temperature 15°C–25°C) were included in the C group (*n* = 80), and those from the southern region (average annual temperature 20°C–28°C, summer temperature 28°C–38°C) were included in the H group (*n* = 45). The inclusion criteria for subjects were the ability to complete the survey correctly, and patients with a history of pregnancy, hysterectomy, and ovariectomy without menarche were excluded. Saliva and serum were collected from all of these individuals and the study population was divided into high-temperature menstrual disorder (HD), high-temperature normal menstrual (HN), control menstrual disorder (CD), and control normal menstrual (CN) group. To better control the variables, key metabolites and regulatory mechanisms of menstrual disorders due to high temperature environment were investigated. Therefore, this paper focuses on the analysis of differential metabolites in women with menstrual disorders in the high-temperature and control groups (HD-CD).

This study was conducted according to the principle of the Declaration of Helsinki. All operational procedures were performed according to ethical principles of the Institute of Environmental and Operational Medicine and approved by the ethics review committee. The subjects were informed about the study’s objective and voluntary participation, and all signed the informed consent form.

### 2.2 Research questionnaire

A total of three questionnaires, including the Menstrual Status Questionnaire, the Influence Factors of Menstrual Questionnaire, and the Symptom Checklist-90 (SCL-90), were administered (Zhang and Zhang, 2013). The Menstrual Status Questionnaire inquires about menstrual characteristics and premenstrual symptoms (the coefficient of internal consistency Cronbach’s *α* = 0.879). The Influence Factors of Menstrual Questionnaire inquires about emotional and psychological status, family history of the disease, medication history, diet, sleep, and environmental conditions (the coefficient of internal consistency Cronbach’s *α* = 0.703). The SCL-90 consists of nine subscale dimensions, namely, Somatization (SOM), Obsessive Compulsive (OC), Interpersonal-Sensitivity (INT), Depression (DEP), Anxiety (ANX), Hostility (HOS), Phobic-Anxiety (PHOB), Paranoid Ideation (PAR), and Psychoticism (PSY). This study was conducted during the summer. All necessary information was collected from the medical records of participants through direct interviews and questionnaires over the last 6 months.

### 2.3 Sample collection and preparation

On the day before saliva collection, all subjects were allowed to only drink water after 21:00 (UTC+8). Subjects’ saliva was collected from 09:00 AM to 11:00 AM according to the saliva collection method specified by (Asai et al. (2018) Subjects were not allowed to drink water, smoke, brush their teeth, perform an energetic exercise, or apply lipstick before saliva collection. Participants’ mouths were rinsed gently with clean water during saliva collection, and straws were used to assist in collecting unstimulated saliva. The collected saliva was stored at −80°C until further use. Furthermore, 5 mL of venous blood was collected on an empty stomach, and serum was collected and stored at −80°C.

### 2.4 Non-targeted saliva metabolomics

#### 2.4.1 Sample pretreatment and analysis

The saliva samples were thawed at 4°C and centrifuged (7,000 *g*, 5 min) to obtain the supernatant. Then, 100 µL of the saliva samples were mixed with 400 µL of precooled methanol acetonitrile solution (1:1, v/v) to remove protein, and then vortex-mixed for 20 min (4°C, 14,000 *g*). The supernatant was freeze-dried and stored at −80°C. For mass spectrometry, 100 μL of acetonitrile aqueous solution (acetonitrile: water = 1:1, v/v) was added to re-dissolution, vortexed, and centrifuged (4°C, 14,000 *g*, 15 min), and the supernatant was collected for analysis.

The procedure was performed in the Agilent 1290 Infinity LC Ultra High-Performance Liquid Chromatography System (UHPLC) Hydrophilic Interaction Liquid Chromatography (HILIC) column. The column temperature was 25°C, the flow rate was .3 mL/min, and the injection volume was 2 μL. The mobile phase comprised A (water +25 mM ammonium acetate +25 mM ammonia) and B (acetonitrile). The gradient elution procedure was as follows: 0–1 min, 95% B; 1–14 min, 95%–65% B; 14–16 min, 65%–40% B; 16–18 min, 40% B; 18–18.1 min, 40%–95% B; and 18.1–23 min, 95% B. The samples were placed in an autosampler at 4°C during the entire analysis.

Electrospray ionization (ESI) experiments were executed on the Triple TOF 5600 mass spectrometer (MS) (AB SCIEX) in positive and negative ion modes. The ESI source conditions after HILIC chromatographic separation were set as follows: ion source Gas 1, 60 psi; ion source Gas 2, 60 psi; curtain gas, 30 psi; source temperature, 600°C; ion spray voltage floating ±5500 V; TOF MS scan m/z range, 60–1000 Da; production scan m/z range, 25–1000 Da; TOF MS scan accumulation time, 0.20 s/spectra; and production scan accumulation time, 0.05 s/spectra. The secondary mass spectrum was acquired using information-dependent acquisition (IDA) and adopting the high sensitivity mode. Declustering potential: ±60 V; collision energy, 35 ± 15 eV; IDA was set as Exclude isotopes within 4 Da, candidate ions to monitor per cycle: 6.

#### 2.4.2 Data analysis

The raw data were converted into mzXML format using Proteo Wizard. The XCMS program (http://xcmsonline.scripps.edu) was adopted for peak alignment, retention time correction, and peak area extraction. MzXML is a file format published by the Institute for Systems Biology, Insilicos, and other companies for the exchange of mass spectrometry data. MzXML offers the advantages of openness, scalability, and flexibility, and it is particularly suitable for storing and exchanging mass spectrometry data. XCMS is a mass spectrometry data analysis software for endogenous metabolites, which provides a complete metabolomics workflow, including signature detection, retention time correction, alignment, annotation, and statistical analysis. The software SIMCA-P 14.1 (Umetrics, Umea, Sweden) was used for pattern recognition, and data were reprocessed by Pareto-scaling; then, multi-dimensional statistical analysis was performed. One-dimensional statistical analysis included Student’s t-test and fold change analysis. Data were visualized using R software. Metabolites with significant differences between the groups (variable importance for the projection (VIP), VIP >1; Wilcoxon rank-sum test *p* < 0.05) were screened to perform qualitative analysis.

### 2.5 Targeted metabolomics of serum neurotransmitters

#### 2.5.1 Sample pretreatment and analysis

The serum samples (100 µL) were mixed with 400 µL of precooled pure acetonitrile containing 1% FA, vortex-mixed, and ultrasonicated in an ice bath for 20 min. The protein was precipitated ultrasonically at −20°C for 1 h in an ice bath, centrifuged (4°C, 14,000 x*g*, 20 min), and taken dry with the supernatant vacuum. For mass spectrometry, 100 μL of ACN/water (1:1, v/v) with 1% FA was added to re-dissolve and centrifuge (4°C, 14,000 x*g*, 20 min), and the supernatant was collected for analysis.

Sample separation was performed using the Agilent 1290 Infinity LC UHPLC system. The mobile phase comprised liquid A (0.1% FA 25 mM ammonium formate aqueous solution) and B (0.1% FA acetonitrile). The sample was placed in an automatic sampler at 4°C, at the column temperature of 45°C, the flow rate of 300 μL/min, and injection volume of 2 μL. The relevant liquid phase gradients were as follows: 0–18 min, 90%–40% B; 18–18.1 min, 40%–90% B; and 18.1–23 min, 90% B. The 5500 QTRAP mass spectrometer (AB SCIEX) was used for mass spectrometry in the negative ion mode. The 5500 QTRAP ESI source conditions were set as follows: source temperature, 550°C; ion source Gas 1, 60 psi; ion source Gas 2, 60 psi; curtain gas, 35 psi; and ion spray voltage floating, 5000 V. The multiple reaction monitoring (MRM) mode was applied to detect the ion pair.

#### 2.5.2 Data analysis

The chromatographic peak area and retention time were measured using the Multiquant software. The neurotransmitter standard was used to correct the retention time and identify the metabolites.

### 2.6 Statistical analysis

SPSS24.0 software was used for statistical analysis. The quality parameters are presented as percentages, and the quality parameters between the two groups were compared using the chi-square test. The influencing factors were analyzed using logistic regression analysis. Wilcoxon rank-sum test was used for comparisons between the two groups. Values with *p* < 0.05 were considered statistically significant.

## 3 Results

### 3.1 Basic information of study subjects

In order to better investigate the effect of high-temperature environment on menstrual disorders in women, some variables were controlled. The recruited women had no smoking or drinking habits and all had the same daily working hours, exercise intensity, and diet structure. A questionnaire was used to compare the basic conditions, including age, BMI, sleep, etc. It was found that anxiety and environmental temperature were significantly different between the two groups, while the remaining relevant variables were no significantly (Table 1, 2 **)**.

**TABLE 1:** Age and BMI of the two groups.

Item	H Group	C Group	*p*
Age	21.42 ± 2.76	22.35 ± 3.09	0.097
BMI	20.76 ± 2.61	20.87 ± 2.54	0.806

**TABLE 2:** Correlation variables of the two groups.

Item	χ^2^	*p*
Medical history	2.666	0.102
Anxiety	6.649*	0.036
Depression	3.875	0.145
Pressure	0.53	0.767
Relationships with colleagues	1.787	0.181
Family history of menstrual disorders	2.542	0.111
Family history of disease	1.753	0.185
Medication history	1.194	0.274
Exercise times	3.853	0.146
Exercise intensity	0.148	0.701
Exercise during menstruation	0.375	0.55
Fatigue	3.387	0.184
Regular diet	0.013	0.91
Dieting	0.079	0.779
Preference for spicy and stimulating flavors	3.146	0.076
Cold drinks and cold food	4.161	0.125
Sleep time	0.159	0.69
Sleep quality	0.796	0.672
The time to fall asleep	4.82	0.09
Environment Temperature	6.828*	0.009

*p* < 0.05, was considered statistically significant.

### 3.2 Menstrual disorders

**FIGURE 1:**
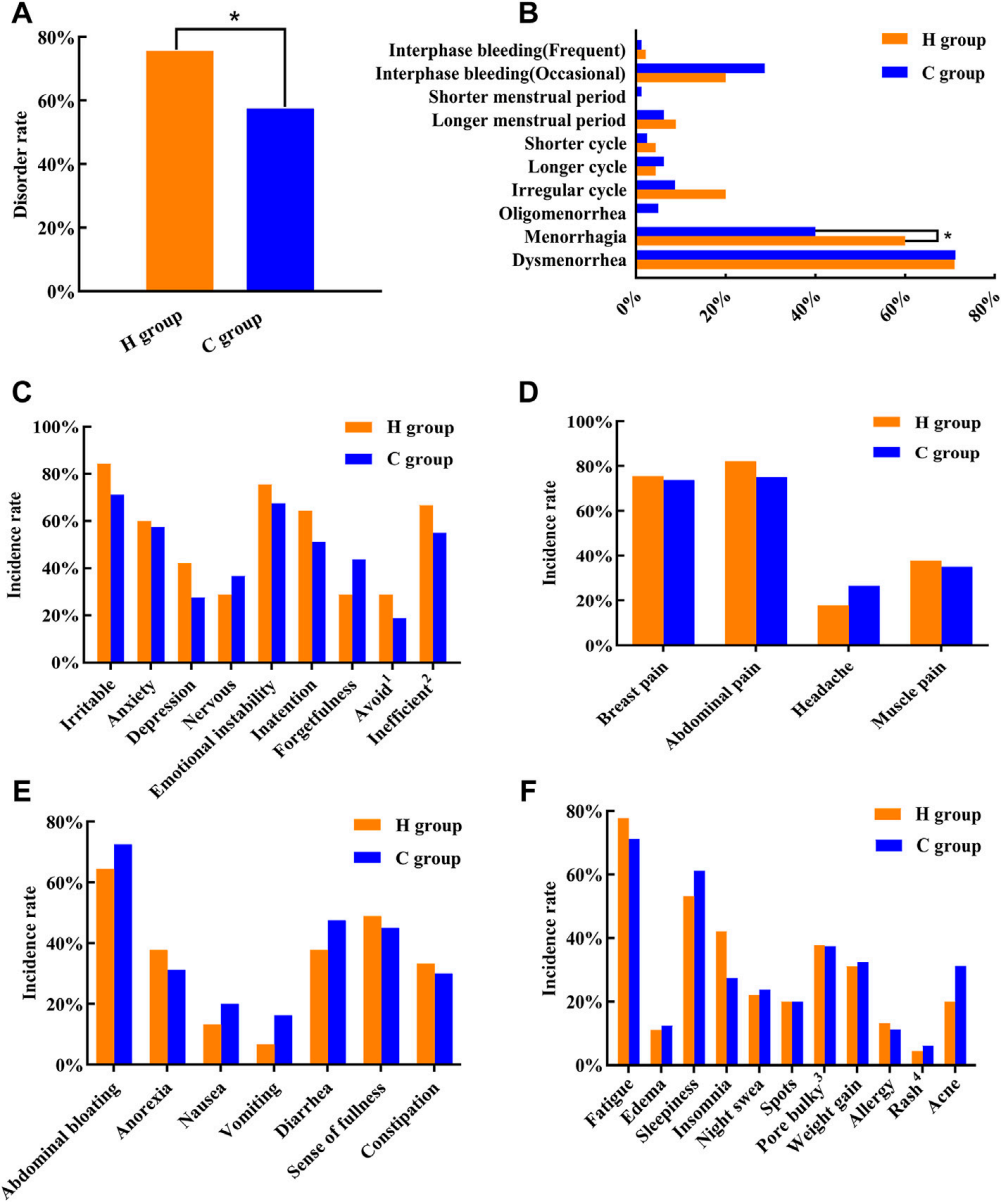
Menstrual disorder status in the two groups of women. **(A)** Menstrual disorder rate. **(B)** Menstrual disorder characteristics. **(C)** Premenstrual symptoms “mood and social functioning.” **(D)** Premenstrual symptoms “physical pain.” **(E)** Premenstrual symptoms “endocrine.” **(F)** Premenstrual symptoms “others.” *p < 0.05, when compared with the C group .1 “Avoid”: a person may show some loneliness and reluctance to meet people during the week before menstruation. 2 “Inefficient”: there is a tendency to do something as well as work less efficiently. 3 “Pore bulky”: some women show enlarged pores on their faces during the premenstrual period. 4 “Rash”: the skin condition that some women experience as a rash on the face before menstruation.

The results of the Menstrual Status Questionnaire revealed that the incidence rate of menstrual disorders in women in the H group was 75.56%, which was significantly higher than that in the C group (57.5%) (*p* < 0.05) (Figure 1A). Thus, exposure to a high-temperature environment may lead to increased incidence of menstrual disorders in women. Based on the results of this survey, we analyzed the characteristics of menstrual disorders and noted that the proportion of women with heavy menstruation was significantly higher in the H group than in the C group (*p* < 0.05) (Figure 1B). Further, we divided the premenstrual symptoms into “emotional and social function” (Figure 1C), “physical pain” (Figure 1D), “endocrine” (Figure 1E), and “others” subgroups (Figure 1F). Analysis using the Chi-square test revealed no significant difference in the premenstrual symptoms between the two groups of women (*p* > 0.05).

### 3.3 Influencing factors of menstrual disorders analysis

The Influence Factors of Menstrual Questionnaire was administered to determine the factors influencing menstrual disturbances in the subjects. Statistically significant variables in the univariate logistic regression served as independent variables, while menstrual disorders served as dependent variables. Multifactorial logistic regression analysis was adopted, which revealed that stress, sleep quality, and temperature feeling acted as the risk factors for menstrual disorders in women in both groups (*p* < 0.05), as can be seen in Table 3. Meanwhile, temperature feeling was analyzed interactively with anxiety, depression, stress, sleep quality, sleep time, and the time to fall asleep, and multiplicative interactions were recorded between temperature feeling and stress, none of which were additive (*p* < 0.05). The study population was stratified by temperature band, and regression analyses were performed separately. In the high-temperature group, univariate and multivariate regression analyses revealed that anxiety, sleep quality, and temperature perception were significant influences on menstrual disorders and that there were no multiplicative or additive interactions between the factors. In the control group, univariate and multivariate regression analyses identified depression as a possible factor influencing menstrual disorders and there were no multiplicative or additive interactions between the factors.

**TABLE 3:** Logistic regression analysis to menstrual disorders and variable in two groups.

Variable	Univariate logistic regression			Multivariate logistic regression	
	Crude OR (95%CI)	*p*		Adjusted OR (95%CI)	*p*
Age (year)	1.019 (0.867, 1.193)	0.814			
BMI (kg/m^2^)	0.897 (0.735, 1.078)	0.260			
Medical history					
No	1				
Yes	3.840 (0.351, 85.024)	0.282			
Anxiety					
1	1			Correlated with other covariables and excluded	
2	8.750 (2.772, 31.932)	<0.001*			
3	24.500 (4.544, 201.188)	0.001*			
Depression					
1	1			No significant and excluded	
2	4.225 (1.505, 12.425)	0.007*			
3	Inf (0.000, Inf)	0.992			
Stress					
1	1			1	
2	3.095 (1.060, 9.987)	0.046*		5.044 (1.191, 28.238)	0.040*
3	13.000 (2.364, 105.812)	0.006*		27.885 (2.328, 606.355)	0.017*
Relationships with colleagues					
1	1				
2	1.114 (0.338, 3.441)	0.853			
Family history of menstrual disorders					
No	1			No significant and excluded	
Yes	4.737 (1.328, 19.559)	0.020*			
Family history of disease					
No	1				
Yes	1.846 (0.071, 47.983)	0.669			
Medication history					
No	1			No significant and excluded	
Yes	3.214 (0.832, 13.740)	0.094*			
Exercise times					
1	1				
2	1.619 (0.581, 4.480)	0.352			
3	4.533 (0.405, 102.073)	0.232			
Exercise intensity					
2	1				
3	0.921 (0.354, 2.437)	0.867			
Exercise during menstruation					
No	1				
Yes	1.599 (0.615, 4.182)	0.334			
Fatigue					
1	1				
2	1.723 (0.542, 6.158)	0.373			
3	1.867 (0.437, 8.345)	0.400			
Regular diet					
No	1				
Yes	0.542 (0.021, 14.074)	0.669			
Dieting					
No	1				
Yes	1.227 (0.154, 7.876)	0.829			
Preference for spicy and stimulating flavors					
No	1				
Yes	2.000 (0.507, 7.913)	0.311			
Cold drinks and cold food					
1	1			No significant and excluded	
2	0.324 (0.061, 1.458)	0.150			
3	0.133 (0.013, 0.964)	0.060*			
Sleep time					
1	1			No significant and excluded	
2	0.383 (0.129, 1.108)	0.077*			
Sleep quality					
1	1			1	
2	3.759 (1.268, 12.304)	0.021*		10.199 (2.180,68.991)	0.007
3	16.917 (3.214, 135.113)	0.002*		45.904 (4.616, 820.710)	0.005*
The time to fall asleep					
1	1			No significant and excluded	
2	2.121 (0.320, 42.122)	0.505			
3	12.444 (1.812, 252.812)	0.028*			
Temperature feeling					
1	1			1	
2	0.885 (0.204, 4.666)	0.875		0.745 (0.082, 8.982)	0.797
3	6.926 (1.674, 36.931)	0.012*		12.031 (1.678, 160.908)	0.027*
Interaction between Temperature feeling and stress				0.137 (0.020, 0.959)	0.045*

Variables with *p < 0.1 in univariate analysis were included in the multivariate analysis, and *p < 0.05 in multivariate analysis was considered to indicate statistical significance.

These results suggested that stress, sleep quality, and temperature feeling acted as the risk factors for menstrual disorders in women in both groups. In the high-temperature group, anxiety, sleep quality, and high-temperature feeling acted as important factors for menstrual disorders, while, in the control group, depression served as an important factor for menstrual disorders because the range of temperature did not include the high temperature and the effect of high temperature could not be assessed. Therefore, in a high-temperature environment, the temperature feeling is an important factor that contributes to menstrual disorders in women.

### 3.4 SCL-90 analysis

To evaluate the recent emotional status of the two groups of women, the SCL-90 was administered, which distinguishes people who already show psychiatric symptoms from those who do not. A higher total score on the SCL-90 indicates a more urgent need for individual intervention. The results of the questionnaire survey and the subsequent analysis by t-test indicated differences in the mental and psychological statuses between the two groups of women; the scores of anxiety and depression were significantly higher in the H group than in the C group (*p* < 0.05). These findings suggest that women living in high-temperature environment are more likely to manifest negative emotions, such as anxiety and depression (Table 4).

**TABLE 4:** The SCL-90 scores of women in both the groups after long-term heat exposure (Mean ± SD).

	H Group	C Group	T	Sig
SUM-SCL	129.44 ± 33.87	113.58 ± 24.19	2.47	0.017*
SOM	1.44 ± 0.56	1.22 ± 0.42	2.05	0.045*
OC	1.56 ± 0.61	1.42 ± 0.52	1.26	0.090
INT	1.44 ± 0.61	1.31 ± 0.52	1.14	0.260
DEP	1.50 ± 0.66	1.19 ± 0.43	2.47	0.018*
ANX	1.32 ± 0.47	1.13 ± 0.37	2.11	0.040*
HOS	1.38 ± 0.55	1.21 ± 0.47	1.61	0.110
PHOB	1.24 ± 0.55	1.04 ± 0.19	2.01	0.050
PAR	1.32 ± 0.47	1.10 ± 0.35	2.43	0.019*
PSY	1.26 ± 0.45	1.10 ± 0.35	1.86	0.070

Compared with the C group, *p < 0.05.

### 3.5 Salivary metabolic profile

**FIGURE 2:**
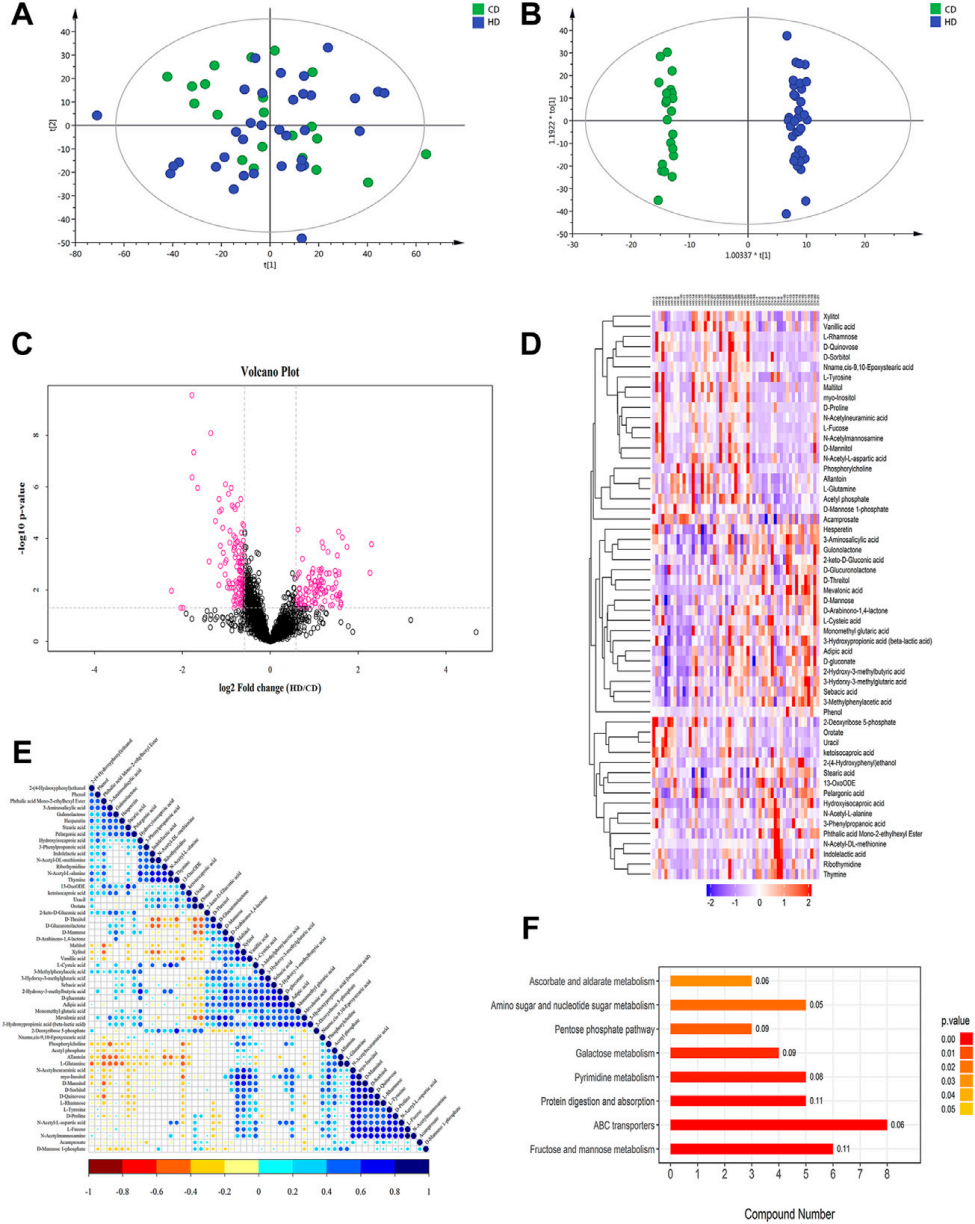
Multivariate statistical analysis, heat map, cluster analysis, and metabolic pathways (take positive ion mode as an example) **(A)** PCA score chart. **(B)** OPLS-DA score chart of saliva metabolite analysis in the HD-CD. **(C)** The volcano map of the differential metabolites in the HD-CD. The red spots in the figure indicate the metabolites with |FC| > 1.5 and *p* < 0.05. These metabolites are the differential metabolites screened by univariate statistical analyses. **(D)** The results of hierarchical clustering of metabolites changed significantly in the sample. Red and blue represent higher and lower metabolite concentrations, respectively. The redder the color, the higher the expression amount of the metabolite. The bluer the color, the lower the expression amount of the metabolite. **(E)** The correlation of metabolites of significant difference in HD-CD. The color shows the correlation between the two metabolites. Blue dots in the graph indicate positive correlations between metabolites, while red dots indicate negative correlations between metabolites. **(F)** The KEGG pathway enrichment analysis results in differential metabolites. Histogram color corresponds to *p*-value, the smaller the *p*-value, statistically more significant is the enrichment of the KEGG pathway (*p* < 0.05). The *X*-axis represents the number of significantly different metabolites were enriched in this pathway, and the value on the histogram is rich factor.

Salivary metabolomics analysis of the study subjects revealed a total of 39 differential metabolites (VIP >1 and *p* < 0.05) in HN-CN (Table 5), and 64 significantly different metabolites were screened in HD-CD (Table 6). We compared HN-CN with HD-CD differential metabolites, and found that 31% of the differential metabolites were duplicated and that the mean fold change of the differential metabolites that were duplicated in both the groups was approximately 1.1, that is, the error caused by the difference between the two populations was small for our target group HD-CD study. This study focused on differential metabolites in HD-CD and did not overlap with HN-CN, which included amino acids, peptides, and carbohydrates that may be involved in the inflammatory responses, immune regulation, amino acid metabolism, and membrane production in an organism. Of the 64 metabolites, the expression levels of 34 salivary metabolites were significantly higher in the HD group than in the CD group: D-proline, Pro-Arg, and phosphorylcholine (PC) expression were 3.54-, 3.53-, and 3.38-times higher in the HD group than in the CD group, respectively (*p* < 0.05). Among the metabolites showing expression, PC is a structural component of various prokaryotic and eukaryotic pathogens with a wide range of immunomodulatory properties (Harnett and Harnett, 1999). Changes in N-acetylneuraminic acid in mammals may trigger inflammation and endocrine diseases as well as cause alterations in inflammatory factors (Reuter and Gabius, 1996). Myo-inositol (MYO) belongs to the family of glycans, which are of high biological importance for maintaining cell membrane stability (Milewska et al., 2016). The expression levels of another 30 salivary metabolites were significantly lower in women in the HD group: mevalonic acid (MVA), phthalic acid mono-2-ethylhexyl ester, and diethyltoluamide expression were 0.29-, 0.52-, and 0.55-times higher than in the CD group, respectively (*p* < 0.05). Among the metabolites showing reduced expression, Tyramine is a biological trace amine that can influence various physiological mechanisms and has certain neuromodulatory properties as well as immunological effects (Andersen et al., 2019). D-mannose is a simple sugar, it has been found to exert an antiinflammatory effect in peripheral diseases (Wang et al., 2021). Correlational analysis of these metabolites identified the possible interactions between these metabolites, such as a positive correlation between MYO and D-sorbitol and a negative correlation between l-glutamine and 3-aminosalicylic acid (*p* < 0.05) (Figure 2E). Taken together, the results of salivary metabolomics suggest that these dysregulated metabolites may play the key role in the development of menstrual disorders in women exposed to high temperatures.

**TABLE 5:** Significant alterations in saliva metabolites of HN-CN.

Name	Description	VIP	Fold change	*p*-value
M139T548	Allantoin	1.996	2.850	0.000
M132T654	Creatine	1.822	2.530	0.008
M130T544	D-Pipecolinic acid	2.322	2.321	0.001
M163T376	L-Rhamnose	1.476	2.126	0.029
M183T493	L-Glutamine	1.905	2.103	0.001
M112T372	Cytosine	1.527	2.051	0.026
M164T427	DL-3-Aminoisobutyric acid	1.501	2.035	0.032
M69T119	Imidazole	1.757	1.945	0.019
M163T396	D-Mannitol	1.721	1.928	0.006
M162T671	L-Carnitine	1.829	1.880	0.033
M181T370	Hydroxyphenyllactic acid	2.128	1.685	0.000
M329T362	Phe-Tyr	1.844	1.653	0.016
M259T941	alpha-D-Glucose 1-phosphate	1.708	1.537	0.028
M231T104	Maleic acid	1.871	1.503	0.006
M180T390	Acamprosate	1.512	1.407	0.042
M147T92	Adipic acid	1.978	1.316	0.028
M96T92	2(1H)-Pyridinone	1.163	0.792	0.024
M149T156	3-Methylphenylacetic acid	1.960	0.753	0.044
M85T172	Isovaleric acid	1.181	0.675	0.015
M141T637	2-Oxoadipic acid	4.145	0.661	0.018
M127T135	Monomethyl glutaric acid	1.035	0.651	0.043
M101T82	Glutaraldehyde	1.249	0.607	0.031
M108T138	Nitrosobenzene	1.010	0.600	0.015
M193T75	Ethyl 3-hydroxybutyrate	1.399	0.597	0.040
M152T82	3-Aminosalicylic acid	1.192	0.582	0.014
M134T769	L-Aspartate	1.230	0.576	0.026
M277T79_2	Phthalic acid Mono-2-ethylhexyl Ester	1.200	0.553	0.008
M255T83	Palmitic acid	1.073	0.536	0.041
M261T429	Ile-Glu	1.009	0.533	0.022
M131T405	Hydroxyisocaproic acid	1.433	0.526	0.000
M303T88	20-Hydroxyarachidonic acid	1.091	0.525	0.026
M158T412	Acetyl-DL-Valine	1.082	0.519	0.012
M317T269	Ribothymidine	1.132	0.508	0.002
M127T141	Thymine	1.139	0.498	0.044
M259T270	Ribothymidine	1.388	0.481	0.009
M220T521	Pantothenate	1.272	0.437	0.031
M130T499	N-Acetyl-L-alanine	1.275	0.430	0.007
M147T555	Mevalonic acid	1.125	0.413	0.025
M125T144	Thymine	3.219	0.386	0.004
M319T126	12-oxo-ETE	1.263	0.360	0.013

**TABLE 6:** Significant alterations in saliva metabolites of HD-CD.

Name	Description	VIP	Fold change	*p*-value
M116T585	D-Proline	1.720	3.545	0.003
M272T867	Pro-Arg	1.918	3.533	0.001
M184T932	Phosphorylcholine	1.039	3.386	0.028
M164T427	DL-3-Aminoisobutyric acid^a^	1.818	3.150	0.006
M308T725	N-Acetylneuraminic acid	1.589	3.062	0.037
M70T585	2-Amino-2-methyl-1,3-propanediol	1.738	3.054	0.003
M173T731	Glycylproline	1.607	3.025	0.023
M114T586	D-Proline	1.517	2.997	0.046
M223T374	D-Quinovose	2.035	2.960	0.002
M163T376	L-Rhamnose^a^	1.815	2.956	0.016
M163T428	L-Fucose	1.748	2.900	0.022
M130T544	D-Pipecolinic acid^a^	2.598	2.762	0.000
M242T895	Phosphorylcholine	1.366	2.366	0.027
M325T785	Maltitol	1.259	2.214	0.047
M155T449	Orotate	1.513	2.151	0.006
M139T548	Allantoin^a^	1.599	2.044	0.007
M179T750	myo-Inositol	1.598	2.002	0.025
M163T198	D-Sorbitol	1.711	1.921	0.042
M111T449	Uracil	1.556	1.903	0.006
M244T701	Pro-Gln	2.040	1.849	0.003
M177T873	Acetyl phosphate	1.753	1.829	0.003
M376T498	(-)-Tylocrebrine	1.358	1.822	0.050
M183T493	L-Glutamine^a^	1.632	1.764	0.011
M129T83	ketoisocaproic acid	1.354	1.733	0.041
M213T817	2-Deoxyribose 5-phosphate	1.551	1.631	0.024
M297T122	Cis-9,10-Epoxystearic acid	1.871	1.611	0.041
M174T765	N-Acetyl-L-aspartic acid	1.706	1.602	0.036
M195T78	4-Hydroxybutanoic acid lactone	1.297	1.576	0.017
M151T468	Xylitol	1.409	1.556	0.029
M259T923	D-Mannose 1-phosphate	1.339	1.520	0.048
M215T494	Val-Pro	1.768	1.477	0.043
M303T512	Phe-His	1.519	1.474	0.037
M261T272	Val-Val	1.659	1.453	0.022
M180T390	Acamprosate^a^	1.616	1.273	0.020
M201T641	Sebacic acid	1.025	0.789	0.042
M157T127	D-Glucuronolactone	1.167	0.775	0.042
M231T520	DL-a-Hydroxybutyric acid	1.789	0.759	0.014
M161T133	D-Mannose	1.282	0.758	0.019
M177T748	D-gluconate	1.405	0.755	0.019
M117T475	2-Hydroxy-3-methylbutyric acid	1.443	0.754	0.031
M133T122	Ethyl 3-hydroxybutyrate^a^	1.419	0.742	0.024
M189T741	N.alpha.-Acetyl-L-lysine	1.201	0.728	0.046
M161T737	L-Gulonic gamma-lactone	1.613	0.724	0.008
M108T138	Nitrosobenzene^a^	1.560	0.722	0.005
M131T405	Hydroxyisocaproic acid^a^	1.447	0.720	0.025
M96T92	2(1H)-Pyridinone^a^	1.737	0.714	0.000
M162T671	L-Carnitine^a^	1.420	0.711	0.041
M152T82	3-Aminosalicylic acid^a^	1.511	0.709	0.002
M161T534	3-Hydorxy-3-methylglutaric acid	1.396	0.686	0.002
M143T146	D-Threitol	1.202	0.678	0.021
M168T701	L-Cysteic acid	1.262	0.663	0.049
M134T452	Oxindole	1.785	0.658	0.004
M125T144	Thymine^a^	2.992	0.640	0.029
M261T429	Ile-Glu^a^	1.576	0.624	0.022
M319T126	12-oxo-ETE^a^	1.315	0.602	0.026
M130T499	N-Acetyl-L-alanine^a^	2.037	0.580	0.005
M149T156	3-Methylphenylacetic acid^a^	6.647	0.575	0.000
M321T88	20-Hydroxyarachidonic acid^a^	1.625	0.572	0.006
M120T272	Tyramine	1.705	0.570	0.000
M85T172	Isovaleric acid^a^	2.127	0.566	0.000
M137T75	2-(4-Hydroxyphenyl) ethanol	1.279	0.561	0.026
M192T65	Diethyltoluamide	1.376	0.551	0.005
M277T792	Phthalic acid Mono-2-ethylhexyl Ester^a^	1.990	0.529	0.000
M147T555	Mevalonic acid^a^	2.437	0.299	0.000

^a^ The repeated metabolites in HD-CD compared with HN-CN, which should not be involved in the development of menstrual disorders.

Based on the LC-MS method, the quality control samples were tightly clustered by the principal component analysis (PCA) model, which showed positive and negative ion patterns, indicating good reproducibility of this project (Figure 2A). The orthogonal partial least squares discriminant analysis (OPLS-DA) model revealed a good clustering and clear differentiation between the groups (Figure 2B). Commonly used univariate analysis methods such as fold change (FC) analysis, t-test, and volcano plot combined the first two analysis to reveal significantly different metabolites between the two groups (Figure 2C). VIP obtained from the OPLS-DA model was used to measure the strength and explanatory power of the expression patterns of each metabolite on the classification of each sample group. Hierarchical clustering of the samples using qualitatively significantly different metabolite expressions revealed that the metabolites that were clustered together had similar expression patterns and were in closer reaction steps during the process of metabolism (Figure 2D).

### 3.6 Enrichment of differential metabolites of the Kyoto Encyclopedia of Genes and Genomes (KEGG) pathway

The KEGG pathway (http://www.kegg.jp/) is a commonly used database for pathway studies. The KEGG pathway is based on the context of a metabolic pathway in which the species or a closely related species are involved. Fisher’s exact test analyzes and calculates the significance level of metabolite enrichment for each pathway to identify the metabolic and signal transduction pathways that are significantly affected. The KEGG pathway enrichment results in the present study showed that the differential metabolites were mainly enriched in eight pathways, namely, ABC transporters, fructose, and mannose metabolism, protein digestion and absorption, amino sugar and nucleotide sugar metabolism, pyrimidine metabolism, galactose metabolism, ascorbate and alternate metabolism, and pentose phosphate pathway (Figure 2F).

### 3.7 Analysis of targeted neurotransmitters

**FIGURE 3:**
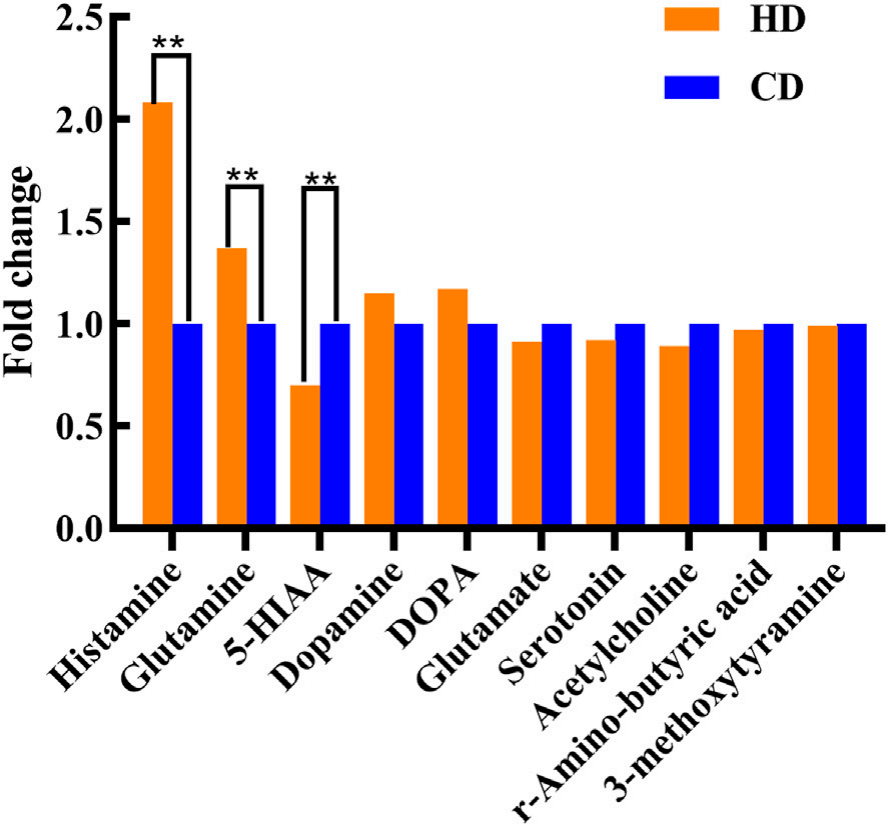
Changes in serum neurotransmitters, as detected by targeted metabolomics analysis. Compared with the CD group, **p < 0.01.

For a more comprehensive exploration of metabolite changes in women with menstrual disorders caused by high-temperature environment, serum was collected from the subjects and analyzed. Based on the MRM approach, targeted metabolomic analysis was performed on serum neurotransmitters in the two groups. No significant differences in neurotransmitters were found in HN-CN. A total of three significant differences neurotransmitters were found in HD-CD. Three neurotransmitters increased expression of histamine (HA) and glutamine, which were 2.077- and 1.366-times higher in the HD group than in the CD group, respectively (*p* < 0.05), and decreased expression of 5-hydroxyindole acetic acid (5-HIAA) in the HD, which was 0.701-times higher than that in the CD group (*p* < 0.05) (Figure 3). The neurotransmitter HA is involved in the neuroendocrine regulation of pituitary hormone secretion and in the regulation of some peripheral hormones. It is critical in the regulation of behavioral state, biological rhythms, body weight, energy metabolism, thermoregulation, fluid homeostasis, stress and reproduction in females. Plasma HA acts as an inflammatory mediator and immune substance (Niaz et al., 2018). 5-HIAA is an important factor associated with human mood, and changes in 5-HIAA are closely related to human mood changes, affecting human endocrine and thus menstrual regulation. This finding suggests that the generation of menstrual disorders in women is associated with inflammatory responses and mood-related neurotransmitters that are altered in a high-temperature environment. This finding indicated that neurotransmitters related to inflammatory response and mood are altered in high-temperature environments.

## 4 Discussion

### 4.1 Menstrual disorders caused by high temperature environment

High temperatures can cause heat stress (Horowitz, 2002; Horowitz, 2007), which is the sum of non-specific responses occurring in humans or animals when an organism is exposed to excessive temperature stimuli exceeding its thermoregulatory capacity in a high-temperature environment. It is a stress factor that special operating populations have to guard against in high-temperature environments (Kovats and Hajat, 2008). During heat stress, the normal thermal homeostasis system of the body may be disrupted, leading to the disruption of digestive somatic functions, impaired blood circulation, and disruption of neuroendocrine functions, which together pose a severe threat to the development of reproductive health concerns in women working under high-temperature environments (Wang et al., 2016; Fan et al., 2019). Menstrual disorders not only affect female reproduction but may also lead to infertility. It can also manifest as hormonal abnormalities, increasing the risk of fatigue, depression, and reducing exercise capacity. Hormonal abnormalities can also have a variety of side effects, such as soft tissue damage that cannot be fully repaired, inhibition of bone formation, and adverse effects on cardiovascular and renal functions. HE can increase the risk of menstrual disorders and affect the reproductive function of women (An et al., 2020). The characteristics of changes and regulatory mechanisms of menstrual disorders that appear at high temperatures remain unclear. We selected two regions with significant differences in their mean temperature and employed a random, whole-group sampling method to conduct a cross-sectional survey of young women belonging to the same-work category. In this study, the confounding factors were controlled accordingly. Only young migrant women, instead of native youth, were sourced from two different regions in this study. In addition, other variables including age, BMI, work intensity, and dietary habits were not significantly different between the two groups. Altitude and environmental temperature were also analyzed as climate variables. It was found that both the regions were in the plains, but there were significant differences in the environmental temperature. Due to the small size of this sample, there was some influence on the analysis of factors, which resulted in large confidence intervals. Based on the analysis of population characteristics and environmental variables, we considered that it is representative of the people in this study, that is, young women who were migrants to both the areas with the same work intensity.

This study found a significantly higher rate of menstrual cycle disorders in young women working in hot working environments. These findings indicate that high-temperature environments are more likely to cause menstrual disorders and increase menstrual volume, but it has no significant effect on premenstrual syndromes. Stress, low sleep quality, and high temperature were noted as the influencing factors that affect menstruation and increase the risk of menstrual disorders in women. Hot environment, as a stressor, has specific effects on the psychology of women working in such environments, mainly in the form of increased negative emotions such as pressure, depression, anxiety, fear, and anger. Common menstrual problems are closely related to increased psycho-emotional changes, anxiety, and excessive psychological stress, which are critical factors affecting menstrual disorders and resulting in amenorrhea (Allsworth et al., 2007; Rafique and Al-Sheikh, 2018). Meanwhile, emotions induce the release of hormones from the pituitary and hypothalamus (Matsuda et al., 1997). Menstruation occurs when there is a change in the regularity of hormones secreted by the ovaries and acting on the endometrium, which is controlled by hormones released by the hypothalamus (Shufelt et al., 2017). In addition, sleep deprivation, short sleep duration, low sleep quality, and altered circadian rhythms inhibit melatonin secretion, affecting ovarian function and reducing the menstrual rate, thereby leading to menstrual disorders or dysmenorrhea in women (Najafi et al., 2018; Czajkowska et al., 2019; Meers and Nowakowski, 2020). In summary, HE may affect women’s emotions and alter their hormone levels, which can interfere with the hypothalamic-pituitary-ovarian (HPO) axis and affect the features of menstruation. These changes induce ovulation and disruption of the menstrual cycle, thereby resulting in menstrual disorders.

### 4.2 Salivary metabolites characteristics of menstrual disorders caused by high-temperature environment

Saliva is an information-rich biofluid that can non-invasively respond to human diseases. We speculate on the possible effects of the substance on the menstrual cycle based on the mechanisms of metabolite action and physiological regulation reported in the literature. To better control variables and find key metabolites of menstrual disorders in women caused by high temperature, this study focuses on HD-CD and there were no repeated with HN-CN. The similarly metabolites are affected by temperature both in populations with and without menstrual disorders, that means that those metabolites should not be involved in the development of menstrual disorders. Among the metabolites with upregulated expression levels, PC is an intermediate in the *in vivo* synthesis of lecithin in animal cells, with a surprising range of immunomodulatory properties that can benefit the infected host by targeting innate and adaptive immune responses. However, its broad immunomodulatory properties can harm the host through immunomodulation (Harnett and Harnett, 1999). The increase of PC in a high-temperature environment indicates that the immune regulation of the human body is activated and the high-temperature environment affects the immune regulation of the human body. N-acetylneuraminic acid, a derivative of neuraminic acid collectively known as sialic acid (SA), is the major SA found in mammalian cells (Varki, 1992). Changes in the SA levels can trigger the development of various diseases, including inflammation, cardiovascular disease, neurological disorders, and endocrine disorders (Reuter and Gabius, 1996). It has also been suggested that elevated SA levels reflect an acute phase response in the inflammatory process and that a positive correlation exists between tumor necrosis factor-α (TNF-α) and interleukin-6 (IL-6), which are the essential mediators of the acute phase response. Moreover, elevated inflammatory factors affect loops such as the HPA axis, which in turn affect reproductive function (Demir et al., 2018). The expression level of N-acetylneuraminic acid was significantly higher in the HD group than in the CD group, indicating an inflammatory response in the organism; thus, elevated inflammatory factors in the body can affect reproduction and lead to the development of menstrual disorders. Therefore, HE may induce inflammatory and immune response modulation, leading to menstrual disorders by affecting the HPA axis loop. MYO and D-sorbitol were mainly enriched in the membrane transport pathway. MYO is widely distributed in nature, and Its derivatives are important components of the cell membrane structural phospholipids and act as precursors of second messengers of the metabolic pathways (Milewska et al., 2016). The results suggested that menstrual cycle disorders are associated with endocrine disruption and maintenance of the cell membrane stability. MYO deficiency and impairment of the MYO-dependent pathways may play crucial roles in the pathogenesis of insulin resistance and hypothyroidism. Insulin and thyroxine are important components of the HPO axis. In a previous study, the kinetic cycle was disturbed and the insulin levels were reduced in female rats after prolonged heat stress exposure (An et al., 2020). Abnormalities in insulin metabolism also underlie several clinical diseases (MacFarlane and Di Fiore, 2018). Heat stress can downregulate the expression of inositol-requiring enzyme 1α (IRE1α), leading to the termination of the IRE1α signaling pathway, which causes an unfolded protein response in cells and affects the production of cell membrane proteins (Homma and Fujii, 2016). Moreover, endocrine regulation and follicular membrane stability are closely related to female reproduction. Therefore, prolonged exposure to heat and hyperthermia may create an imbalance in the metabolism of insulin and thyroxine. When the expression of MYO is dysregulated, it affects the formation of follicular membranes and results in endocrine disorders that affect the female reproductive function and the menstrual cycle. The expression of D-sorbitol, a metabolite that was positively correlated with MYO, was also increased. Appropriate D-sorbitol supplementation can significantly reduce the formation of blastocysts and increase the apoptosis index (Lin et al., 2015). When the expression of D-sorbitol increases in a hot environment, the possibility of germ cell apoptosis increases, which affects the reproductive function and leads to menstrual disorders. Therefore, long-term exposure to high temperatures and HE may result in an imbalance in the metabolism of insulin and thyroxine. Cell membrane stability is reduced, which may increase apoptosis and cause menstrual disorders.

Among the metabolites with reduced expression in this study, Tyramine is a biological trace amine that is generated through decarboxylation of the amino acid tyrosine, and substantial evidence suggests that tyramine is a neuroactive chemical exhibiting multiple physiological effects (Lange, 2009); It can also affect the various physiological mechanisms, exhibits neuromodulatory properties, and cardiovascular and immunological effects (Andersen et al., 2019), stimulates the insulin-IGF-1 signaling (IIS) pathway, and blocks the induction of stress response genes by activating adrenergic-like receptors in the intestine. Tyrosine can directly or indirectly act on the ovaries to inhibit luteal function, thereby affecting reproduction. D-mannose diminishes the proinflammatory response and boosts the antiinflammatory response. *In vitro* experiments with different monosaccharides further confirmed that only d-mannose treatment blocked macrophage phagocytosis in a dose-dependent manner. As phagocytosis of myelin debris has been known to increase inflammation, decreasing phagocytosis could result in decreased activation of proinflammatory macrophages (Wang et al., 2021). Also, Mannose can decrease bacterial attachment to the uterine mucosa in mares (King et al., 2006). Humoral factors belonging to the innate immune system such as mannose-binding lectin seem to be associated with pregnancy outcome probably by modifying the level of inflammation at the feto-maternal interface, and mannose-binding lectin is involved in the maintenance of an inflammatory environment in uterus (Vianna et al., 2010; Christiansen, 2013). Therefore, changes in the N-acetylneuraminic acid levels, MYO, and tyramine in salivary metabolites at a high-temperature environment may affect the menstrual cycle through inflammatory responses, influence membrane production, and participate in immune regulation, leading to the development of menstrual disorders.

In this study, KEGG pathway enrichment revealed the involvement of several metabolites in carbohydrate metabolism, mainly fructose and mannose, galactose, and amino and nucleotide sugar metabolism. Glucose metabolism is the core component of energy metabolism, essential in the maintenance of normal physiological functions of the body. Abnormal glucose metabolism is closely associated with metabolic syndrome and diseases such as cancer (Andersen et al., 2013). In addition, abnormal glucose metabolism is associated with several gynecological disorders (Ferreira and Motta, 2018). Insulin resistance, as a pathogenic base of glucose metabolism abnormalities, affects the action of sex hormones on the ovaries and endometrium through insulin-like growth factor-1 receptor (IGF-1R), leading to anovulation or endometrial lesions (Li et al., 2012). Therefore, abnormal glucose metabolism triggers gynecologically related diseases also induces menstrual disorders.

### 4.3 Serum neurotransmitters characteristics of menstrual disorders caused by high-temperature environment

A neurotransmitter is a chemical “messenger” molecule that transmits signals between synapses. The secretion of neurotransmitters promotes the balance of amino acid metabolism in the body, regulates the body’s immune functions and cardiovascular activities, and mediates smooth muscle contraction. In this study, serum-targeted metabolomics revealed an increased expression of HA in the HD group, which was 2.077-times higher than that in the CD group. HA is present in the mammalian myocardium, mast cells, basophils, skin, gastrointestinal tract, and lungs, as well as in the central nervous system. Central HA, as a central neurotransmitter, is related to obesity, diabetes, and endocrine disorders (Watanabe and Yanai, 2001). Generally, HA has a stimulatory but indirect effect on the release of these hormones through the activation of postsynaptic receptors in the hypothalamic regions. Histaminergic neurons appear to be involved in the mediation of stress-induced release of ACTH, β-END, α-MSH, and PRL. An increase in HA also causes an increase in the plasma GnRH, LH, and FSH hormone levels, which affect the regulation of the HPA axis, which in turn also affects reproduction, leading to menstrual disturbances (Niaz et al., 2018). Plasma HA is mainly used as an inflammatory mediator and immune substance. Past studies have confirmed that HA released by mast cells can stimulate HA type 2 receptor (H2R) in the rat kidney as an inflammatory mediator. The release of renin and stressful conditions increase the HA levels in the hypothalamus and the periphery systems in mice (He et al., 2009). This observation is consistent with those of our saliva metabolomics analysis, in which inflammatory reactions occurred under high-temperature environments. In contrast, the expression of 5-HIAA, which is a primary end product of 5-hydroxy tryptamine (5-HT) metabolism and plays an emotional regulation role, decreased under a high-temperature environment (Elghozi and Laude, 1989). The dysfunction of 5-HT can cause different mental diseases, including depression, impulsive aggression, and high pressure, which is also consistent with our previous questionnaire results. The results revealed that thyrotropin-releasing hormone (TSH) has a significant negative correlation with 5-HIAA (Strawn et al., 2004). Therefore, prolonged exposure to high temperatures may increase anxiety and stress in humans and affect the endocrine system, which in turn may lead to menstrual disorders in women.

### 4.4 Comprehensive analysis of menstrual disorders caused by high temperature environment

Non-targeted salivary metabolomics provides a comprehensive and systematic analyses of the results of endogenous metabolic activities in women living in a high-temperature environment. The metabolomics reveal changes in the metabolite levels in the menstrual disorder state. Targeted neurotransmitter metabolomics can further complement the non-targeted metabolic results. The analysis of salivary metabolomics results in this study suggested that menstrual disorders were associated with inflammatory responses and immune regulation. The expression of N-acetylneuraminic acid was found to be significantly increased in the salivary metabolites in HD compared with CD, and this difference was associated with some inflammatory factors (Varki, 1992; Demir et al., 2018). The elevated expression of N-acetylneuraminic acid reflected the acute phase of the inflammatory response, which is an important manifestation of the inflammatory response of an organism. The reduced expression of D-mannose is also an important manifestation of immunomodulatory functions occurring in response to inflammation. Through targeted neurotransmitter metabolomics, this study investigated whether changes in the organism are consistent with changes in their saliva or whether there is some intrinsic correlation between them. Neurotransmitter studies revealed significant alterations in the neurotransmitters with immunomodulatory functions and mood regulation. HA is mainly involved in the immune regulation in the peripheral blood; its expression was significantly elevated, which is consistent with the salivary metabolomics results reported previously (He et al., 2009). The present results further demonstrated that immune regulation may be the reason for menstrual disorders caused by the high-temperature environment. In addition, the 5-HIAA expression was found to be reduced in the high-temperature group and the 5-HT exhibited mood-regulating effects, which can lead to depression and anxiety. This finding is also consistent with the results of questionnaire survey. In our study, the administration of SCL-90 questionnaire revealed that women in the high-temperature group were more likely to be anxious and depressed relative to those in the control group. The literature also suggests that a high-temperature environment can influence human psychological emotions and lead to increased development of negative emotions (Lee et al., 2018). These results imply that the increased rate of menstrual disorders in women living in a high-temperature environment is related to inflammatory responses, immune regulation, and possibly an increase in the negative female emotions that affect the endocrine system and cause menstrual disorders. Therefore, changes in the serum neurotransmitter levels provide better corroboration of salivary metabolomic results, which lays the foundation for the exploration of the mechanisms of menstrual disorders in women caused by living in a high-temperature environment. This study has limitations in sample selection and control for confounding factors, especially the influence of food on metabolism. In order to minimize the impact of diet on saliva metabolism, the subjects were administered according to uniform standards and their daily meals were organized according to China’s nutritional dietary recommendations. Although they have the same diet structure, the types of food are difficult to be completely consistent. Therefore, although we tried to control sample selection, work and living habits to a certain extent, this study is still not perfect and has limitations. We hope that the samples can be better controlled to obtain better results in future studies.

## 5 Conclusion

These findings revealed that the rate of menstrual disorders increased in women who were exposed to a long-term heat environment, and stress and anxiety were identified as the main influencing factors. The changes in the metabolite levels, such as the levels of N-acetylneuraminic acid, MYO, and tyramine may be candidate markers for early diagnosis. The elevation of the N-acetylneuraminic acid level could respond to the acute-phase response during an inflammatory process, which affects the reproductive system by influencing the HPA axis loop. Myo-inositol causes the termination of the IRE1α signaling pathway by inducing the downregulation of IRE1α protein, which is required for inositol, in response to heat stress, thereby resulting in the formation of an unfolded protein in the cell reaction, which in turn affects oocyte membrane production. Moreover, decreased tyramine, with changes in the complexing concentrations, can act directly or indirectly on the ovary to inhibit the luteal functions, which in turn affects reproduction. These changes in the differential metabolites may be closely related to the occurrence of menstrual disorders in women. The effect of high temperature as a stressor on mood is closely related to the occurrence of menstrual cycle disorders. Therefore, it is suggested that a high-temperature environment and mood control may reduce the risk of female reproductive health.

## Data Availability

The raw data supporting the conclusions of this article will be made available by the authors, without undue reservation.

## Supplementary material

Supplementary Table S1 Metabolomics analysis date(HD-CD).

Supplementary Table S2 Metabolomics analysis date(HN-CN).

Supplementary Datasheet S1 Research questionnaires.

## Glossary

H group: high-temperature group
C group: control group
HD: high-temperature menstrual disorder group
HN: high-temperature normal menstrual group
CD: control menstrual disorder group
CN: control normal menstrual group
5-HIAA: 5-hydroxyindole acetic acid
HE: Heat exposure
SCL-90: Symptom Checklist-90
SOM: Somatization
OC: Obsessive Compulsive
INT: Interpersonal-Sensitivity
DEP: Depression
ANX: Anxiety
HOS: Hostility
PHOB: Phobic-Anxiety
PAR: Paranoid Ideation
UHPLC: Ultra High-Performance Liquid Chromatography
HILIC: Hydrophilic Interaction Liquid Chromatography
ESI: electrospray ionization
MS: mass-spectrometer
IDA: information dependent acquisition
VIP: variable importance for the projection
MRM: multiple reaction monitoring
QC: quality control
PCA: principal component analysis
OPLS-DA: Orthogonal partial least squares discriminant analysis
FC: fold change
MVA: mevalonic acid
MYO: myo-inositol
FET: Fisher’s Exact Test
KEGG: Kyoto Encyclopedia of Genes and Genomes
HA: histamine
HPO: Hypothalamus-pituitary-ovaries
SA: sialic acid
TNF-α: tumor necrosis factor-α
IL-6: interleukin-6
HPA: hypothalamic–pituitary–adrenal
IRE1α: inositol-requiring enzyme 1α
UPR: unfolded protein response
IIS: insulin-IGF-1 signaling
IR: Insulin resistance
IGF-1R: insulin-like growth factor-1 receptor
H2R: histamine type 2 receptor
5-HT: 5-hydroxy tryptamine.
