# Supplementary material for: Characteristics of menstrual cycle disorder and saliva metabolomics of young women in a high-temperature environment
Source: Front Physiol. 2023 Jan 13;13:994990. doi: 10.3389/fphys.2022.994990 (PMC9880290; doi:10.3389/fphys.2022.994990)
Supplement: Supplementary file 2 [file DataSheet1.PDF]

Questionnaire 1 Menstrual Status

NO. ________ Age __________ Height ___________
Weight ________ Work years __________ Degree of education ___________

Menstrual status

1. Menstrual status
   Regular ( ) Disturbed ( ) Amenorrhea ( )
2. Abnormal bleeding during the menstruation
   No ( ) Frequent ( ) Occasional ( )
3. Days of menstruation (days)
   2-7 ( ) < 2 ( ) > 7 ( ) Irregular ( )
4. Menstrual cycle (days)
   21-35 ( ) < 21 ( ) > 35 ( ) Irregular ( )
5. Menstrual flow (total number of sanitary napkins used during menstruation) (pieces)
   5-15 ( ) < 5 ( ) > 15 ( )
6. Dysmenorrhea
   Never ( ) Mild ( ) Severe ( ) Unbearable pain ( )

Premenstrual symptoms

1. Irritable
   Never ( ) Light ( ) Severe ( )
2. Anxiety
   Never ( ) Light ( ) Severe ( )
3. Depression
   Never ( ) Light ( ) Severe ( )
4. Nervous
   Never ( ) Light ( ) Severe ( )
5. Emotional instability
   Never ( ) Light ( ) Severe ( )
6. Inatention
   Never ( ) Light ( ) Severe ( )
7. Forgetfulness
   Never ( ) Light ( ) Severe ( )
8. Avoid[1]
   Never ( ) Light ( ) Severe ( )
9. Inefficient[2]
   Never ( ) Light ( ) Severe ( )
10. Breast pain
    Never ( ) Light ( ) Severe ( )
11. Abdominal pain
    Never ( ) Light ( ) Severe ( )
12. Headache
    Never ( ) Light ( ) Severe ( )
13. Muscle pain

Never ( ) Light ( ) Severe ( )

14. Abdominal bloating
    Never ( ) Light ( ) Severe ( )
15. Anorexia
    Never ( ) Light ( ) Severe ( )
16. Nausea
    Never ( ) Light ( ) Severe ( )
17. Vomiting
    Never ( ) Light ( ) Severe ( )
18. Diarrhea
    Never ( ) Light ( ) Severe ( )
19. Abdominal fullness
    Never ( ) Light ( ) Severe ( )
20. Constipation
    Never ( ) Light ( ) Severe ( )
21. Fatigue
    Never ( ) Light ( ) Severe ( )
22. Edema of the extremities
    Never ( ) Light ( ) Severe ( )
23. Sleepiness
    Never ( ) Light ( ) Severe ( )
24. Insomnia
    Never ( ) Light ( ) Severe ( )
25. night sweat
    Never ( ) Light ( ) Severe ( )
26. Spots
    Never ( ) Light ( ) Severe ( )
27. Pore bulky[3]
    Never ( ) Light ( ) Severe ( )
28. weight gain
    Never ( ) Light ( ) Severe ( )
29. Allergy
    Never ( ) Light ( ) Severe ( )
30. Rash[4]
    Never ( ) Light ( ) Severe ( )
31. Acne
    Never ( ) Light ( ) Severe ( )

1 “Avoid”: a person may show some loneliness and reluctance to meet people during the week before menstruation.

2 “Inefficient”: there is a tendency to do something as well as work less efficiently.

3 “Pore bulky”: some women show enlarged pores on their faces during the premenstrual period.

4 “Rash”: the skin condition that some women experience as a rash on the face before menstruation.

Questionnaire 2 Influence Factors of Menstrual

NO.____________

Please tick (√) in the corresponding position according to your (last six months) actual situation and personal feelings

General status

1. Menstrual status
   Regular ( ) Disturbed ( ) Amenorrhea ( )
2. Age at menarche
   < 10 years old ( ) 11-17 years old ( ) > 17 years old ( )
   No natural menstruation ( )
3. Marital and reproductive status
   Unmarried ( ) Married ( ) Number of children ( )
4. Disease History
   None ( ) Irregular menstruation ( ) Polycystic ovary syndrome ( )
   Benign breast diseases (such as breast hyperplasia, benign fibroids of the breast) ( )
   Pelvic inflammatory disease ( ) Hyperthyroidism ( ) Hypothyroidism ( )
   Obesity ( ) Hypertension ( ) Diabetes mellitus ( ) Hyperlipidemia ( )
   Heart disease ( ) Constipation ( ) Others ( )

Daily psychological and emotional (including menstruation)

1. Anxiety
   Never ( ) Mild ( ) Severe ( )
2. Depression
   Never ( ) Mild ( ) Severe ( )
3. Stress
   Never ( ) Mild ( ) Severe ( )
4. Relationship with colleagues
   Good ( ) General ( ) Bad ( )

Family history of disease and medication

1. Family history of menstrual disorders
   Yes ( ) No ( )
2. Family history of disease (multiple choice)
   None ( ) Irregular menstruation ( ) Polycystic ovary syndrome ( )
   Benign breast diseases (such as mastopexy,, benign fibroids of the breast) ( ) Pelvic inflammatory disease ( ) Hyperthyroidism ( ) Hypothyroidism ( ) Obesity ( ) Hypertension ( ) Diabetes mellitus ( ) Hyperlipidemia ( ) Heart disease ( ) Constipation ( ) Others ( )
3. Have you taken any medication for menstruation?
   No ( ) Yes ( ) Drug name ( )
4. Have you taken hormonal drugs?

No ( ) Yes ( ) Drug name( )

Life Habits

1. Training times per week (> 30min into statistics)
   > 5 times ( ) 3-5 times ( ) < 3 times ( )
2. The intensity of exercise
   Low ( ) Medium ( ) High ( )
3. Have you ever do exercise during menstruation?
   Yes ( ) No ( )
4. Do you feel fatigued ?
   Never ( ) Light ( ) Severe ( )
5. Are you eating regularly?
   Yes ( ) No ( )
6. To diet
   Yes ( ) No ( )
7. Consumption of cold drinks or ice-cream per week (times)
   < 3 ( ) 3-5 ( ) > 5 ( )
8. Sleep duration
   < 6 hours ( ) 6-8 hours ( ) > 8 hours ( )
9. Sleep quality
   Good ( ) General ( ) Bad ( )
10. Sleep disorder
    Yes ( ) No ( )
11. Bedtime
    Earlier than 10PM ( ) 10-11PM ( ) 11PM-12AM ( ) 0-1AM ( )
12. How does the local temperature feel?
    Cold ( ) Moderate ( ) Hot ( )

Questionnaire 3 Symptom Checklist-90 (SCL-90)

NO.____________

Please read each one carefully. After you have done so, please choose the option which best describes how much that problem has bothered or distressed you during the past 1 week including today. All questionnaires will be treated confidentially!

0 = not at all 1 = a little bit 2 = moderately 3 = quite a bit 4 = extremely

1 Headaches ( )
2 Nervousness or shakiness inside ( )
3 Unwanted thoughts or ideas that won't leave your head ( )
4 Faintness or dizziness ( )
5 Loss of sexual interest or pleasure ( )
6 Feeling critical of others ( )
7 The idea that someone else can control your thoughts ( )
8 Feeling others are to blame for most of your troubles ( )
9 Trouble remembering things ( )
10 Worried about sloppiness or carelessness ( )
11 Feeling easily annoyed or irritated ( )
12 Pains in heart or chest ( )
13 Feeling afraid in open spaces or on the street ( )
14 Feeling low in energy or slowed down ( )
15 Thoughts of ending life ( )
16 Hearing voices that other people do not hear ( )
17 Trembling ( )
18 Feeling that most people cannot be trusted ( )
19 Poor appetite ( )
20 Crying easily ( )
21 Feeling shy or uneasy with the opposite sex ( )
22 Feeling of being trapped or caught ( )
23 Suddenly scared for no reason ( )
24 Temper outbursts that you could not control ( )
25 Feeling afraid to go out of your house alone ( )
26 Blaming yourself for things ( )
27 Pains in lower back ( )
28 Feeling blocked in getting things done ( )
29 Feeling lonely ( )
30 Feeling blue ( )
31 Worrying too much about things ( )
32 Feeling no interest in things ( )
33 Feeling fearful ( )
34 Your feelings being easily hurt ( )

35 Other people being aware of your private thoughts (　　)
36 Feeling others do not understand you or are unsympathetic (　　)
37 Feeling that people are unfriendly (　　)
38 Having to do things very slowly (　　)
39 Heart pounding or racing (　　)
40 Nausea or upset stomach (　　)
41 Feeling inferior to others (　　)
42 Soreness of your muscles (　　)
43 Feeling that you are watched or talked about by others (　　)
44 Trouble falling asleep (　　)
45 Having to check and double check what you do (　　)
46 Difficulty making decisions (　　)
47 Feeling afraid to travel on buses, subways or trains (　　)
48 Trouble getting your breath (　　)
49 Hot or cold spells (　　)
50 Having to avoid certain things, places or activities (　　)
51 Your mind going blank (　　)
52 Numbness or tingling in parts of your body (　　)
53 A lump in your throat (　　)
54 Feeling hopeless about the future (　　)
55 Trouble concentrating (　　)
56 Feeling weak in parts of your body (　　)
57 Feeling tense or keyed up (　　)
58 Heavy feelings in your arms or legs (　　)
59 Thoughts of death or dying (　　)
60 Overeating (　　)
61 Feeling uneasy when people are watching or talking about you (　　)
62 Having thoughts that are not your own (　　)
63 Having urges to beat, injure or harm someone (　　)
64 Awakening in the early morning (　　)
65 Having to repeat the same actions such as touching, counting, washing (　　)
66 Sleep that is restless or disturbed (　　)
67 Having urges to break or smash things (　　)
68 Having ideas or beliefs that others do not share (　　)
69 Feeling very self-conscious with others (　　)
70 Feeling uneasy in crowds such as shopping or at a movie (　　)
71 Feeling everything is an effort (　　)
72 Spells of terror or panic (　　)
73 Feeling uncomfortable about eating or drinking in public (　　)
74 Getting into frequent arguments (　　)
75 Feeling nervous when you are left alone (　　)
76 Others not giving you proper credit for your achievements (　　)
77 Feeling lonely even when you are with people (　　)
78 Feeling so restless you couldn’t sit still (　　)

79 Feeling of worthlessness (　　)
80 Feeling that familiar things are strange or unreal (　　)
81 Shouting or throwing things (　　)
82 Feeling afraid you will faint in public (　　)
83 Feeling that people will take advantage of you if you let them (　　)
84 Having thoughts about sex that bother you a lot (　　)
85 The idea that you should be punished for your sins (　　)
86 Feeling pushed to get things done (　　)
87 The idea that something serious is wrong with your body (　　)
88 Never feeling close to another personal (　　)
89 Feelings of guilt (　　)
90 The idea that something is wrong with your mind (　　)
